# Quantitative Detection of Pharmaceuticals Using a Combination of Paper Microfluidics and Wavelength Modulated Raman Spectroscopy

**DOI:** 10.1371/journal.pone.0123334

**Published:** 2015-05-04

**Authors:** Derek Craig, Michael Mazilu, Kishan Dholakia

**Affiliations:** University of St. Andrews, Department of Physics and Astronomy, St. Andrews, Fife, United Kingdom; CNR, ITALY

## Abstract

Raman spectroscopy has proven to be an indispensable technique for the identification of various types of analytes due to the fingerprint vibration spectrum obtained. Paper microfluidics has also emerged as a low cost, easy to fabricate and portable approach for point of care testing. However, due to inherent background fluorescence, combining Raman spectroscopy with paper microfluidics is to date an unmet challenge in the absence of using surface enhanced mechanisms. We describe the first use of wavelength modulated Raman spectroscopy (WMRS) for analysis on a paper microfluidics platform. This study demonstrates the ability to suppress the background fluorescence of the paper using WMRS and the subsequent implementation of this technique for pharmaceutical analysis. The results of this study demonstrate that it is possible to discriminate between both paracetamol and ibuprofen, whilst, also being able to detect the presence of each analyte quantitatively at nanomolar concentrations.

## Introduction

Recently, paper based sensing has emerged in the field of point of care testing with applications in the area of biosensing, environmental monitoring and food quality control.[1–3] Paper provides a means by which microfluidic devices can be fabricated in a very low-cost, simple and reproducible manner. Patterning of paper using techniques such as ink-jet and wax printing produce defined hydrophilic channels in the paper structure. These control the flow of liquid through the sensor.[4,5] Due to the inherent wicking capability of paper, the passive transport of liquid through pre-defined channels is possible and a vast range of chemicals have been shown to be compatible with the paper substrate.[6] As a result of these key physical properties, paper microfluidics has emerged as a promising complementary technique to current microfluidic technologies with the key advantage of not requiring significant external instrumentation (e.g. microfluidic pumps) to function. Paper microfluidics has the promise to truly realise a lab on a chip (rather than a chip in a lab) device due to the approach being fast, simple to implement as well as offering ease of transport and disposal. Although there are significant promising advantages of paper microfluidics there remain a number of limitations such as poor accuracy and sensitivity that constrain their applications.[6,7] Currently, a number of detection techniques are being explored to overcome such disadvantages. These include colorimetric, electrochemical and fluorescent detection technologies.[8–10] Powerful optical approaches such as Raman spectroscopy confer the possibility of label free detection of analytes on paper microfluidic devices. However, this technique in its native form has so far been obviated in favour of surface enhanced Raman scattering (SERS), which has been employed in a small number of instances. To date the notable uses of SERS in this field include the quantitative detection of narcotics, such as cocaine and heroin, and the development of ELISA type formats to detect antigen-antibody interactions.[11–13] These examples have been successful in achieving the detection of analytes down-to nanomolar concentrations. The fabrication of these substrates rely on the depositing of an enhancement material, such as nanoparticles or nanorods, onto the paper substrate.[14] However, there are challenges to employing this technique, such as, difficulty in achieving a uniform covering of the paper substrate with the enhancing material and the loss of key functionalities such as separation and pre-concentration on the paper device.[15] Most importantly the reproducibility achieved using SERS can be highly variable.[14,16] Attempts to exert control over this enhancement have led to different techniques being introduced to improve the reproducibility and fabrication of the SERS substrates. These include ink jet printing and vapour deposition techniques.[17,18] Herein, we describe the first demonstration of Raman spectroscopy on paper microfluidics without resorting to surface-enhancement, as a very promising alternative technique to overcome these current disadvantages. In particular we use wavelength modulated Raman spectroscopy for our work which overcomes serious drawbacks, particularly related to the paper fluorescence. Our approach is inherently simple and powerful, and yields quantitative information from the device. Indeed, Raman spectroscopy is based on the inelastic scattering of light from a sample. The resulting spectrum of the scattered photons reflects a shift in frequency characteristic of specific vibrational modes of the analyte being interrogated. As a result of this, a fingerprint spectrum is obtained from which individual analytes can be detected. In this way, multiple analytes can be distinguished simultaneously.[19–21] Raman spectroscopy is regarded as a powerful, widely applicable analytical technique, which lends itself to the identification of unknown compounds.[22–24] The integration of Raman spectroscopy with other optofluidic devices has previously been explored with significant success. However, the signal obtained from Raman scattering is typically weak due to only 1 in 10^6^ photons being Raman scattered.[25] As a result the weak Raman signal can be obscured due to auto-fluorescence from the substrate or the sample being analysed.[26] Numerous techniques have been employed to suppress background fluorescence including time resolved Raman spectroscopy and shifted excitation Raman difference spectroscopy (SERDS).[27,28] The alternative, simple technique that we employ here is wavelength modulated Raman spectroscopy (WMRS).[29] WMRS involves recording a series of Raman spectra, which are slightly shifted in excitation wavelength (<1 nm) with respect to one another. The significant difference between WMRS and SERDS is that the WMRS technique employs multiple excitation wavelengths, which results in a significant increase in the signal to noise ratio (S/N) of the spectra obtained.[29–31] In standard Raman spectroscopy the bands shift in accordance with the shift in excitation wavelength yet the fluorescence is insensitive to small shifts in wavelength and as a result remains constant. Using multivariate, principal components analysis (PCA) the modulated Raman information can then be recovered and the fluorescent signal eliminated from the Raman signal. Herein, we describe the use of WMRS to eliminate the inherent background fluorescence from the paper substrate, which permits the identification of pharmaceutical analytes, which have been swabbed onto the paper microfluidic device. This enables us to identify individual analyte components and realise a notable increase in the S/N.

## Experimental

### Paper device preparation

The paper devices were designed in the Microsoft PowerPoint software package and bulk printed onto A4 Whatman No. 1 filter paper using a Xerox ColorQube 8570 DN solid wax printer. Once the devices had been printed they were heated at 150°C to disperse the wax through both sides of the paper to create the 3D channels desired. After heating, the devices were cut to size (length: 2.5 cm, width 1.5 cm) and allowed to cool prior to being used. The paper devices are produced using the procedure shown in S1 Fig.

### Swabbing procedure

Each of the pharmaceuticals were diluted to the required concentration using purified MilliQ water. Of the resulting solution 10 mL were deposited into a 50 mL plastic sampling tube. The solution was swabbed by fully immersing the paper device three times in the solution prior to analysis. In order to ensure each of the paper devices were exposed to the solution for an equal amount of time, each device was immersed in the corresponding solution for 10 seconds three times prior to subsequent analysis by WMRS. This ensured that each device was fully covered by the immersion solution.

### Raman spectroscopy setup

Modulated Raman spectra were acquired using a system whose details are reported elsewhere[29]. The system is based upon a tunable Littman geometry diode laser (Sacher Lasertechnik, centre wavelength of at λ = 785 nm, maximum power 1 W, total tuning range 200 GHz). Laser tuning was controlled with a waveform/function generator (Keithley, 50 MHz) that modulated the wavelength. A telescope enlarged the size of the laser beam to fill the back aperture of a microscope objective (Olympus, magnification 40x/NA = 0.74) subsequent to passage through a line filter. The inelastically scattered Raman photons were collected through the same objective and coupled through a F/# matcher to a spectrometer with a 400 lines/mm grating. Detection was performed with a deep depletion, back illuminated and thermo-electrically cooled CCD camera (Newton, Andor Technology). Uniform illumination of the sample was realised with a standard Kohler illumination set-up in transmission mode.

## Results and Discussion

**Fig 1:**
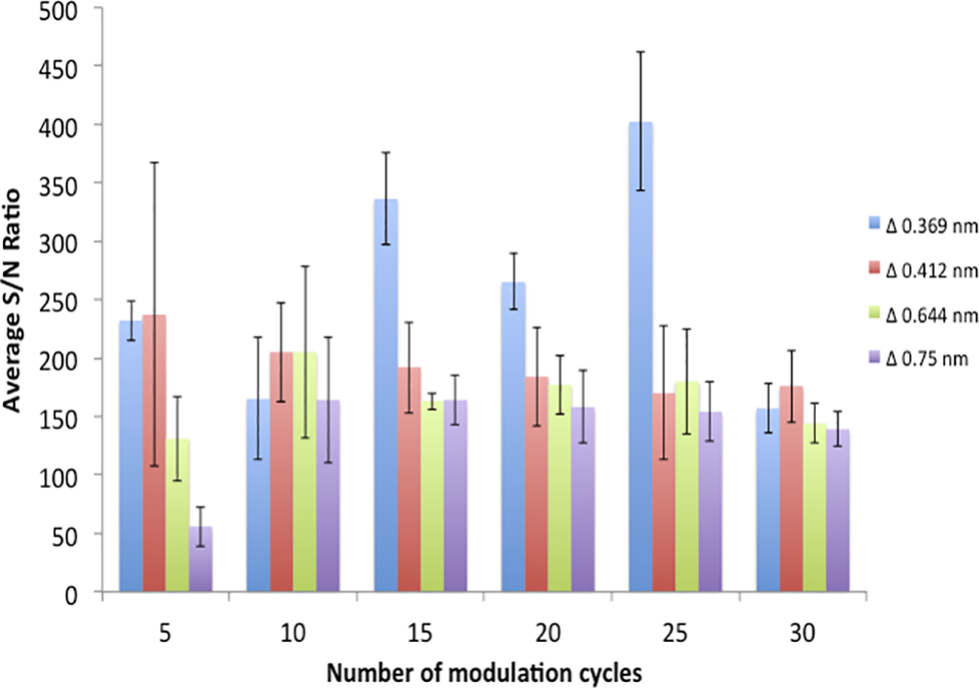
WMRS measurements of the signal to noise ratio (S/N) of the cellulose band at 1089 cm^-1^. Bar chart shown represents measurements of the S/N using a 4s exposure time whilst varying the number of kinetic cycles and band-to-band voltage. Error bars shown are the standard deviation of 5 measurements.

The optimisation of WMRS has previously been discussed by Mazilu *et al*.[32,33] Briefly, the optimal conditions for WMRS required an optimisation of a number of factors including; the modulation amplitude, the time constant used for a single spectral acquisition, the sampling rate across one modulation cycle and the number of modulation cycles which are performed per experiment.[32] The standard Raman spectra of a single unmodified paper device showed a number of Raman bands were present which were assigned to the various stretches and bending modes of C-C and C-H cellulose bands. The most intense band detected occurred at 1089 cm^-1^. To optimise the WMRS conditions the S/N was calculated using the intensity of this band and the standard deviation of the Raman free region as noise. The S/N was monitored as each individual set of conditions was modified. A summary of these results is shown in Fig 1.

Measuring changes in the S/N based upon the alteration of these parameters highlights a number of factors contribute simultaneously to its optimisation. Three different exposure times were tested (data shown in S1 Table) and the 4-second exposure time provided the most consistent and highest S/N achievable. As the number of modulation cycles was incrementally increased from 5 to 30 cycles, the S/N became more consistent, however, this prolonged the time required to perform the analyses. When only five modulation cycles were used significant deviations in the S/N were observed.

**Fig 2:**
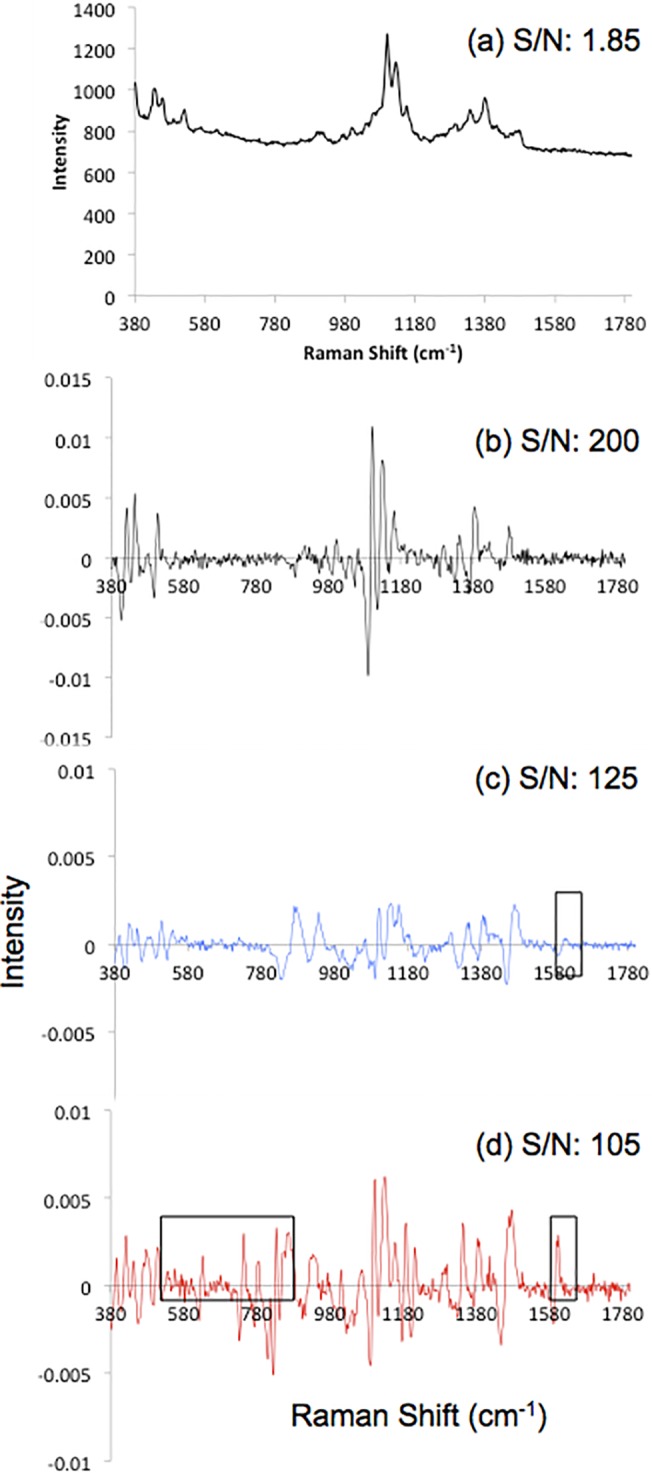
Spectra of the paper microfluidic device before and after swabbing of pharmaceuticals. (a) Standard Raman spectra of paper device and (b)-(d) WMRS spectra of paper only, paper + paracetamol and paper + ibuprofen respectively. S/N quoted are measured for the 1089 cm ^-1^ band and are an average of 10 spectra of each individual sample.

Therefore, a compromise was made to gain a consistent S/N over the shortest period and the number of modulation cycles was assessed to be optimum at 15. Another key variable was the amplitude of the modulation cycle. Four different wavelength modulation amplitudes were explored and each was found to provide an improvement in the S/N in comparison to the standard Raman spectrum. The S/N obtained for each of the amplitudes tested indicated that no significant enhancement of S/N was gained when the amplitude was greater than Δ**λ** = 0.37 nm without resulting in increased statistical errors occurring between the measurements performed i.e. increase in the standard deviations of the average S/N measurements obtained. Therefore, the highest achievable S/N over the shortest number of modulation cycles occurred when using a modulation amplitude of Δ**λ** = 0.37 nm, with 15 modulation cycles and 4s exposure time. These optimum conditions were then implemented for all WMRS experiments in this study. The significant enhancement of the S/N gained from the implementation of these conditions has been shown in Fig 2.

The difference in S/N between the standard Raman spectrum of the paper device, Fig 2(A), and the WMRS spectrum of the paper device, Fig 2(B), is over 100 fold. As well as the increase in the S/N, the significant fluorescence background of the paper substrate was removed through this process. Thus, the distinctive features of the fingerprint spectrum of the paper could easily be identified.

Following the implementation of the optimised parameters for WMRS, real time analysis was performed to test if the detection of pharmaceuticals on paper microfluidic devices was possible. Typical detection mechanisms require colorimetric or electrochemical changes using current techniques. The additional advantage of WMRS coupled with paper microfluidics is the ability to identify key vibrational bands related to the spectrum of each individual component. The key point that enables our study, as shown in Fig 2, is that we may discriminate between the WMRS spectra obtained from the blank paper device, Fig 2(B), and the devices used to swab the paracetamol (c) and ibuprofen samples (d) respectively. As shown in Fig 2(C) a distinctive band arises at 1600 cm^-1^, which can be assigned to the amide-stretching band for paracetamol. Distinctive bands can also be detected for the ibuprofen sample in Fig 2(D) with bands arising between 550 and 800 cm^-1^, which are distinctive to the ibuprofen spectra. A further band arises at 1590 cm^-1^, which can be assigned, to the carboxyl group-stretching mode of ibuprofen. Although the paracetamol spectra displays an identifiable band difference from the paper substrate and ibuprofen, the differences in spectral position and intensity are minimal. Therefore, to improve on this experimental analysis it is required to employ further data analysis methods to enhance the discrimination between the paper substrate and the analytes, swabbed on the paper surface.

**Fig 3:**
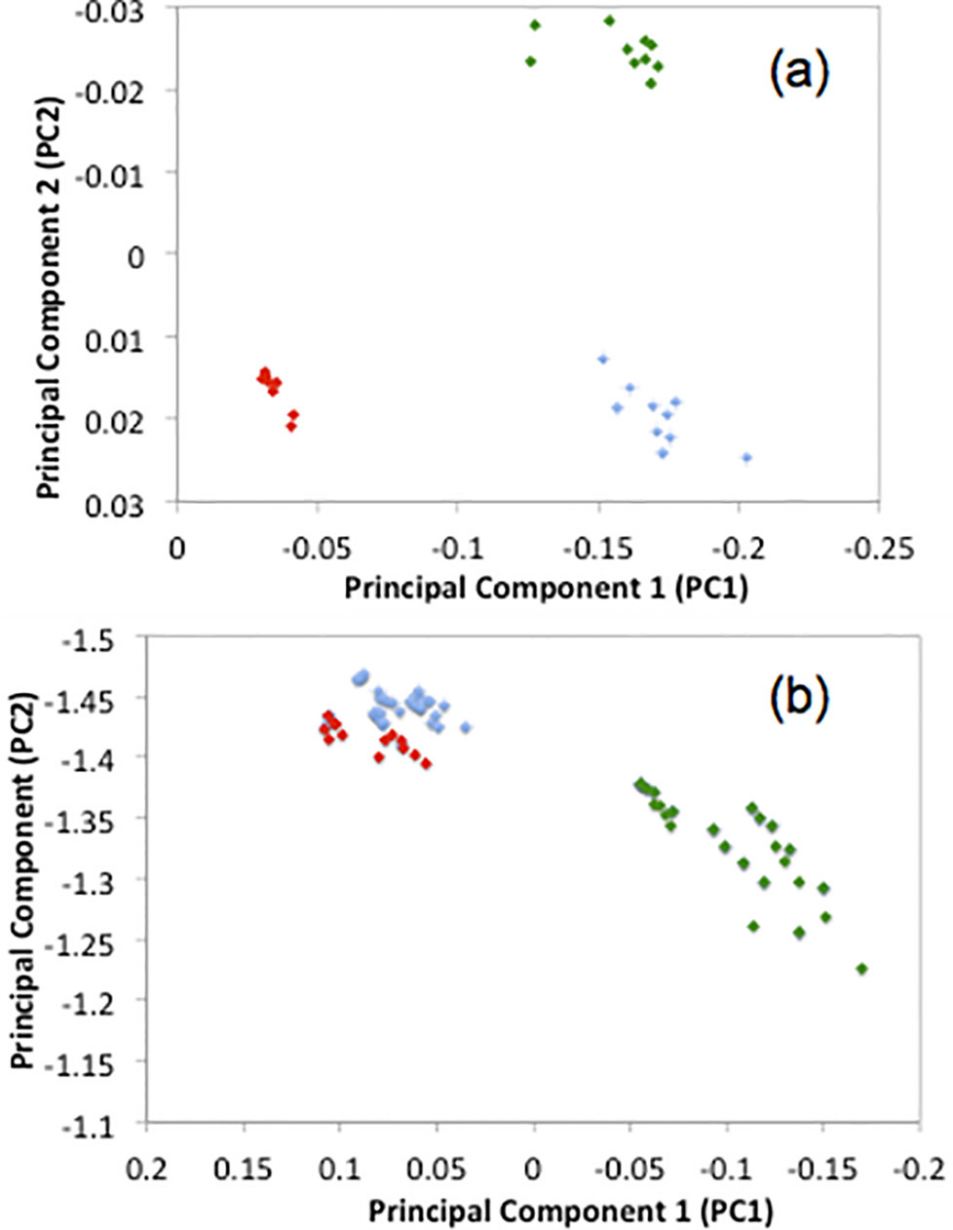
Principal component analysis of (a) WMRS study of paper microfluidics device (green), as well as paper + paracetamol (blue) and paper + Ibuprofen (red). (b) Standard Raman study of paper microfluidics device, as well as paper + paracetamol and paper + Ibuprofen.

Principal component analysis (PCA) is a dimensionality reduction procedure, which accounts for the variance within sets of observed data measurements. PCA scatter plots shown in the subsequent figures show the first two principal components, which demonstrate the greatest variance between samples. Analysis was performed over multiple spectra of all three types of sample using both standard Raman spectroscopy and WMRS and the resulting data analysis is shown in Fig 3.

As shown in Fig 3, the standard Raman spectroscopy analysis results in the production of three data clusters for each of the analytes examined. However, the clusters for both the paracetamol and ibuprofen are not adequately separated. As a result of this, it is not possible to conclusively separate all three components using standard Raman spectroscopy as shown in Fig 3(A). In comparison, the use of WMRS provides a higher discrimination allowing us to identify the individual analyte. We observe distinctive clusters being formed with adequate separation, thus ensuring there is no overlap in the spectral analysis, in turn allowing us to conclusively identify each analyte.

**Fig 4:**
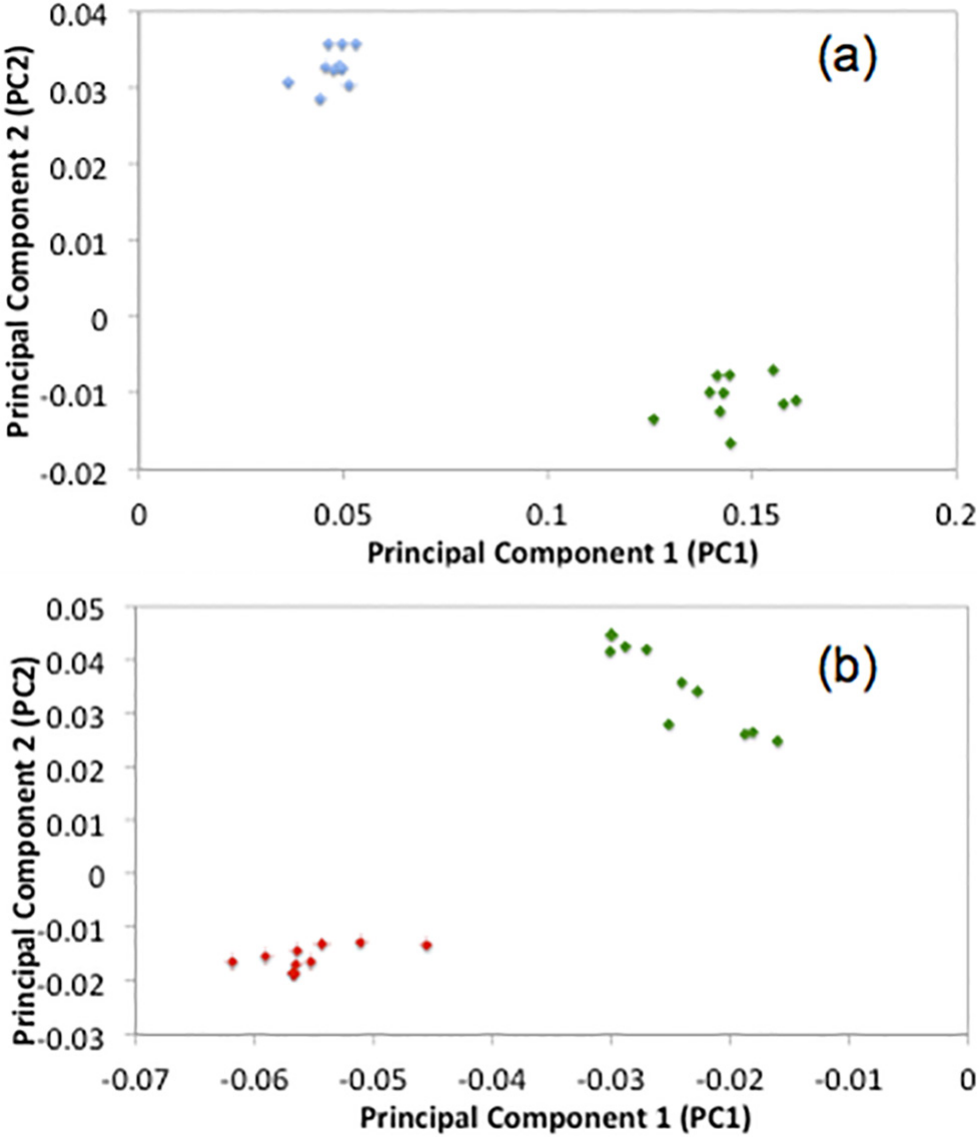
PCA scatter plot of, PC2 vs. PC1, for analysis of (a) paper and paracetamol (blue) vs. the paper device (green) only and (b) paper and ibuprofen (red) vs. paper device only.

Further, a limit of detection study was performed to experimentally identify the lowest concentration of both paracetamol and ibuprofen detectable on the paper substrate. By serial dilution, a range of concentrations of both paracetamol and ibuprofen were produced. Using the swabbing method and the optimised WMRS conditions detailed previously, it was possible to achieve adequate cluster separation of both components down-to nanomolar concentrations. The PCA figures showing cluster separation for paracetamol and ibuprofen on the paper substrate are shown in Fig 4(A) and 4(B) respectively. Fig 4 demonstrates that it is possible to separate the data for the pharmaceuticals using the paper microfluidic device qualitatively when concentrations in the nanomolar range were analysed.

**Fig 5:**
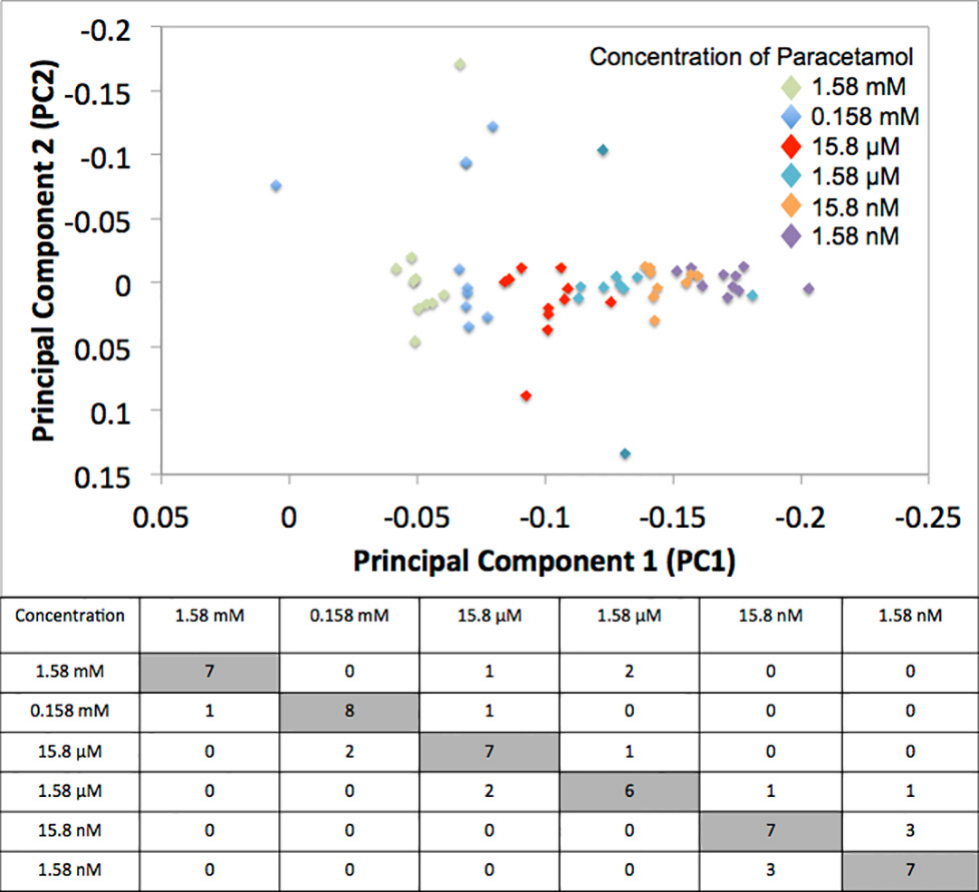
PCA scatter plot, PC2 vs. PC1, for analysis of varied concentrations of paracetamol on individual paper devices. Table shows the confusion matrix from PCA analysis of a limit of detection study of paracetamol on paper microfluidic devices. Numbers indicate the overlap of data points between each concentration studied.

To demonstrate quantitative analysis, a range of concentrations of both paracetamol and ibuprofen were swabbed onto individual paper devices and analysed by WMRS. The PCA scatter plots and the resulting confusion matrix are shown in Fig 5, where it can be observed that following the classification experiment it was possible to segregate each individual concentration of paracetamol. The quantitative identification of individual ibuprofen concentrations was also achieved and this data is found in S2 Fig.

The confusion matrix (Fig 5) shown employs the leave one out method. The diagonal of the matrix represents the number of diluted samples that can be correctly identified and attributed to their correct concentration. Ideally this number would be 10 to represent the fact that the 10 replicate analyses for each concentration had produced data with little inherent variance. However, although this number is not obtained, the confusion matrix shows that the majority of the data clusters together correctly without any significant variance being present. The matrix also highlights that there remains a challenge to improve upon the data acquired with greater variance being generated, as the concentration of paracetamol is sequentially decreased.

## Conclusions

This study demonstrates the first use of WMRS in combination with paper microfluidics for the real-time detection of multiple analytes simultaneously. The use of WMRS for this application establishes that the common sensitivity issues which plague conventional detection techniques used with paper microfluidics can be overcome, with sensitivity of analyte detection being achieved in the nanomolar range. As a result of the success of this study it is possible to determine an experimental limit of detection for paracetamol and ibuprofen at concentrations of 1.58 nM and 96.8 nM respectively, when using a combination of WMRS and paper microfluidics. This level of sensitivity is at least equal with the current examples of SERS based paper microfluidic detection yet this methodology does not require a prolonged fabrication process and is not hindered by substrate reproducibility. Based upon this data, WMRS could play a pivotal role in the integration of paper microfluidics for a range of analyte detection fields with increasing sensitivity.

## Supporting Information

S1 Fig Preparation of paper microfluidic device for swabbing. (i) Device was designed using Microsoft Powerpoint prior to being printed using a Xerox 8850DN solid wax printer. (ii) the device is printed onto an A4 sheet of Whatman No.1 filter paper, (iii) the sheet of filter paper is then heated to 150°C for 2 minutes to re-distribute the wax through both sides of the paper. (iv) From the A4 sheet of devices, single devices are then obtained in preparation for swabbing of the pharmaceutical compounds.
(TIF)

S2 Fig PCA scatter plot, PC2 vs. PC1, for analysis of varied concentrations of ibuprofen on individual paper devices. Table shows the confusion matrix from PCA analysis of a limit of detection study of paracetamol on paper microfluidic devices. Numbers indicate the overlap of data points between each concentration studied.
(TIF)

S1 Table Measured S/N as the number of modulation cycles (5–30) and modulation amplitude (Δλ) were altered for different exposure times: (a) 3s exposure time, (b) 4s exposure time and (c) 5s exposure time. Measurements are an average of 5 replicates.
(TIF)
