# Supplementary figures and images for: Quantitative Detection of Pharmaceuticals Using a Combination of Paper Microfluidics and Wavelength Modulated Raman Spectroscopy
Source: PLoS One. 2015 May 4;10(5):e0123334. doi: 10.1371/journal.pone.0123334 (PMC4418578; doi:10.1371/journal.pone.0123334)

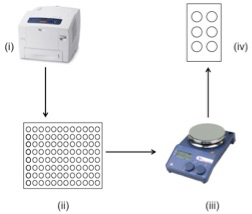

Supplement: S1 Fig — (i) Device was designed using Microsoft Powerpoint prior to being printed using a Xerox 8850DN solid wax printer. (ii) the device is printed onto an A4 sheet of Whatman No.1 filter paper, (iii) the sheet of filter paper is then heated to 150°C for 2 minutes to re-distribute the wax through both sides of the paper. (iv) From the A4 sheet of devices, single devices are then obtained in preparation for swabbing of the pharmaceutical compounds. (TIF) [file pone.0123334.s001.tif]

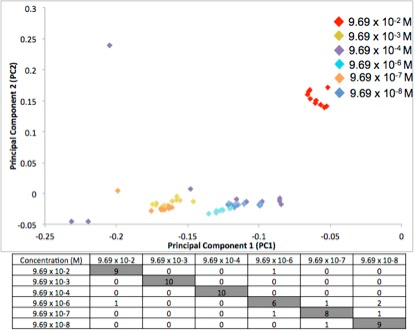

Supplement: S2 Fig — Table shows the confusion matrix from PCA analysis of a limit of detection study of paracetamol on paper microfluidic devices. Numbers indicate the overlap of data points between each concentration studied. (TIF) [file pone.0123334.s002.tif]

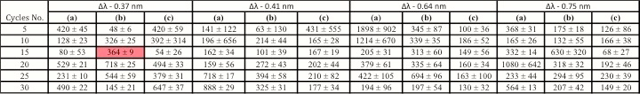

Supplement: S1 Table — Measurements are an average of 5 replicates. (TIF) [file pone.0123334.s003.tif]
